# Automatic computer science domain multiple-choice questions generation based on informative sentences

**DOI:** 10.7717/peerj-cs.1010

**Published:** 2022-08-16

**Authors:** Farah Maheen, Muhammad Asif, Haseeb Ahmad, Shahbaz Ahmad, Fahad Alturise, Othman Asiry, Yazeed Yasin Ghadi

**Affiliations:** 1 Department of Computer Science, National Textile University, Faisalabad, Pakistan; 2 Department of Computer, College of Science and Arts in Ar Rass, Qassim University, Ar Rass, Qassim, Saudi Arabia; 3 Department of Information Technology, College of Computing and Information Technology at Khulais, University of Jeddah, Jeddah, Saudi Arabia; 4 Department of Computer Science/Software Engineering, Al Ain University, Abu Dhabi, UAE

**Keywords:** BERT, Multiple choice questions, Natural language processing, Text analysis, TF-IDF

## Abstract

Students require continuous feedback for effective learning. Multiple choice questions (MCQs) are extensively used among various assessment methods to provide such feedback. However, manual MCQ generation is a tedious task that requires significant effort, time, and domain knowledge. Therefore, a system must be present that can automatically generate MCQs from the given text. The automatic generation of MCQs can be carried out by following three sequential steps: extracting informative sentences from the textual data, identifying the key, and determining distractors. The dataset comprising of various topics from the 9th and 11th-grade computer science course books are used in this work. Moreover, TF-IDF, Jaccard similarity, quality phrase mining, K-means, and bidirectional encoder representation from transformers techniques are utilized for automatic MCQs generation. Domain experts validated the generated MCQs with 83%, 77%, and 80% accuracy, key generation, and distractor generation, respectively. The overall MCQ generation achieved 80% accuracy through this system by the experts. Finally, a desktop app was developed that takes the contents in textual form as input, processes it at the backend, and visualizes the generated MCQs on the interface. The presented solution may help teachers, students, and other stakeholders with automatic MCQ generation.

## Introduction

Since its introduction in the mid-20^th^ century, multiple choice questions (MCQs) have been considered a practical approach among the various assessment methods. For instance, MCQs have been extensively used for educational assessment, market research, and elections. More precisely, the underlying assessment criteria is a simple way to test candidates’ knowledge in less time. Moreover, for self-assessment, MCQs-based assessment is also a convenient tool for evaluators, since it is easy to mark MCQs. Furthermore, the division of marks is comfortable and straightforward in the MCQs-based assessment method. MCQs based assessment gained popularity with the advent of data preprocessing machines and scanners since these machines made it possible to check many questions within no time (Mitkov, 2003).

**Figure 1:**
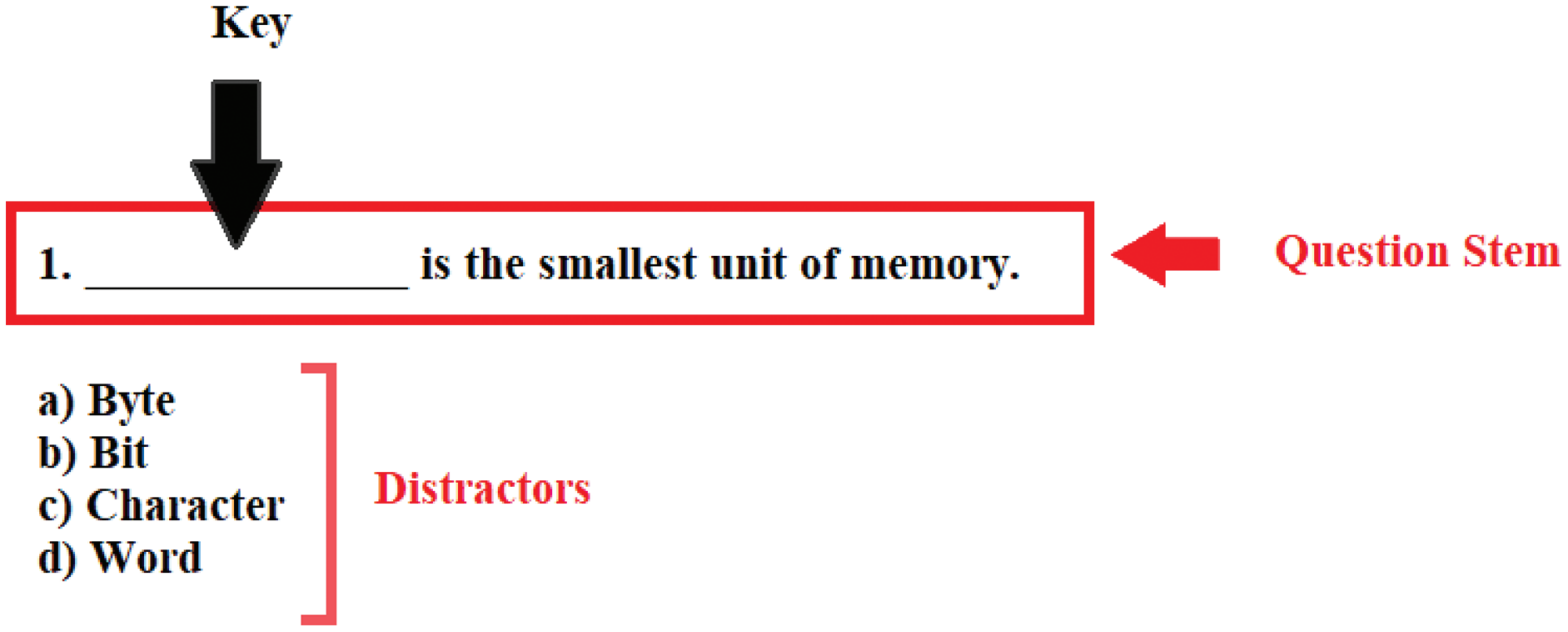
Structure of MCQ.

MCQ generation is a difficult task for humans since it requires domain knowledge and understanding of pedagogical processes with context and tone. It is challenging for exam setters to prepare MCQs with a suitable statement and relevant options manually, since every sentence in the text cannot be a candidate for MCQ. Moreover, reading the entire topic and extracting essential lines or concepts for MCQ generation is a time-consuming task. Furthermore, making statements concise without changing the context and choosing options is tricky. MCQ-based questions comprises three parts, the stem, key, and distractor, as depicted in Fig. 1. The stem is the question or statement that inquires something, the key is the central word/concept that is asked, and distractors are similar options, including the correct answer. In case not prepared adequately by the setter, reading the stem and making a decision about the appropriate key among distractors may take plenty of time (Papasalouros, Kanaris & Kotis, 2008). Hence, it is required that a system may be presented that can develop the MCQs intelligently.

**Figure 2:**
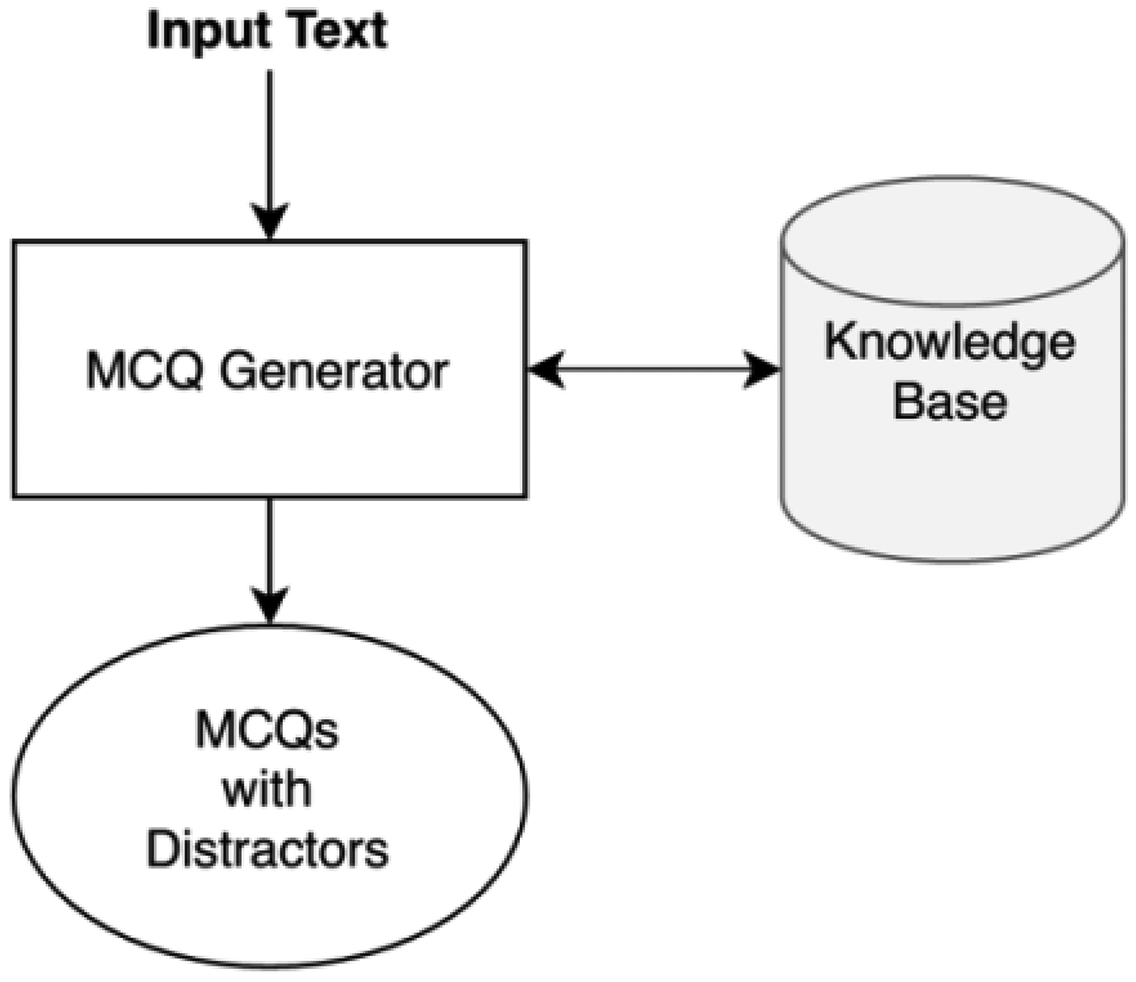
Input and output of the system.

With the enhancement of automation, we are handing off every task to machines. Most advancements have become possible due to machine learning (ML), natural language processing (NLP), and other related tools. Similar to other domains, researchers are trying to automatically generate the assessment tests, including question answers, filling in the blanks, MCQs, *etc*. Automatic MCQ generation is getting popular; many tests are being taken online and making MCQ generation fast and efficient. Since automatic MCQ generation may help the teachers and the students with efficient assessments and active learning. We present a system that automatically makes MCQs for computer science topics using NLP and ML tools. The system takes raw text consisting of computer science based topics. The system’s output comprises of MCQs based on imperative stems of the given topic. Each MCQ consists of a stem with a key and four distractors consisting of one correct answer and three wrong relevant answers. Figure 2 shows the inputs and output of our system (Genest & Lapalme, 2011).

In detail, the presented MCQ generation system can generate MCQs of stems containing quality phrases (Liu et al., 2015). Moreover, MCQs are generated based on informative stems containing most of the domain literature’s core knowledge and terminologies. However, most of the models are trained or use only natural languages corpora and WordNet, where words are conceived as their natural meaning, *e.g*., cloud (the collection of water vapors), but if we take cloud as computer science terminology, it depicts a different meaning and context, *i.e.*, (groups of servers or data centers, where data is kept or services are provided *via* the internet), the same concept runs with the windows and mouse. Additionally, MCQs are generated from unstructured data. Furthermore, stem selection is improved by obtaining an extractive summary of the given text, so we extract the imperative knowledge from the entire topic. Finally, an interface is presented as a front end that takes the unstructured text of the employed domain and outputs effective MCQs in a structured way. In detail, for extractive text summarization to find the informative sentences and key phrases, the bidirectional encoder representations from transformers (BERT) model for generating text embeddings and K-means clustering are employed. A text summary provides the sentences that give the main idea and concept of the whole topic and automatically discards less helpful information. This helps to get the sentences closest to the centroid for creating a summary. The sentences are then scored on several features like quality phrases (Liu, Shang & Han, 2017; Tahir et al., 2021), TF-IDF (Wu et al., 2008), number of nouns and verbs, number of stop words, and Jaccard similarity of chapter title with candidate stems. Since readily available systems for generating MCQs lack informative sentences, the quality of generated distractors is low. The proposed system overcomes such gaps in the prevalent works (Malinova & Rahneva, 2016; Susanti et al., 2016; Satria & Tokunaga, 2017).

### Introduction to informative sentences

Informative sentences give knowledge about an important concept or imperative information. The extraction of informative sentences is done by extractive summarization and scoring using quality phrases, TF-IDF, *etc*. The importance of summarization and quality phrases is discussed in the following subsections.

#### Summarization

Text summarization methods belong to two types, abstractive and extractive. Abstractive summarization is closest to the way humans generate a summary. Abstractive summarization usually extracts the text’s key points and rephrases it, including the vocabulary beyond the specified text, and it is smaller in size (Genest & Lapalme, 2011). Undoubtedly, many researchers are working on abstractive summarization as it is closer to the human way of summarization, which is advantageous. But it requires a massive human summarization dataset for complex algorithms and deep learning, rules with restricted generalizability, and training of several GPUs over many days for automatic generation of summery. At the same time, extractive summarization creates a summary containing actual phrases and the same sentence structure from the source data. The proposed system only considers such text-based stems mined through extractive text summarization.

#### *Quality* phrases

A phrase contains more information than a word, so our system requires a phrase mining technique to extract quality phrases from the underlying domain. Similar works have been done previously using N-gram (Tahir et al., 2021) and topical phrase mining techniques. In detail, the N-gram technique facilitates identification and extracts frequent N-grams from the given text (Tahir et al., 2021). Similarly, topical phrase mining is a helpful technique for phrase mining, topic identification, social event discovery, *etc*. (Li et al., 2018). Another work proposes a knowledge discovery method for an information retrieval system to extract informative and most frequent phrases (Aljuaid et al., 2021). In Papasalouros, Kanaris & Kotis (2008), the researchers employ the online frequent sequence discovery method to extract frequent phrases. TF-IDF (Wu et al., 2008) and KEA (Zhu et al., 2013) are text analysis techniques for calculating raw frequency in a given text *corpus*. But these techniques calculate the raw frequency of phrases based on frequent pattern mining without considering their semantic meanings. For the extraction of quality phrases based stems from the extractive summary, a quality phrase mining technique (Liu et al., 2015) extracts semantically meaningful phrases instead of frequent patterns from raw text.

The quality phrase mining technique counts the word’s frequency, analyzes phrases semantically, and recognizes the quality phrases by considering some features. For example, quality phrase mining techniques make decisions based on features like concordance, completeness, informativeness, and popularity (Liu et al., 2015).

### Introduction to distractors

Distractors are “options” in MCQs. The proposed system provides four distractors containing one correct answer and three related but wrong answers. The system makes distractors by following steps:

- Searching words relevant to key
- Create a list of distractors
- Choosing random words from the list

In this system, distractors are made by using a lexical database, “WordNet” (Miller, 1995), online resource wiktionary (https://en.wiktionary.org/), and “google search” results.

#### Introduction to WordNet

**Figure 3:**
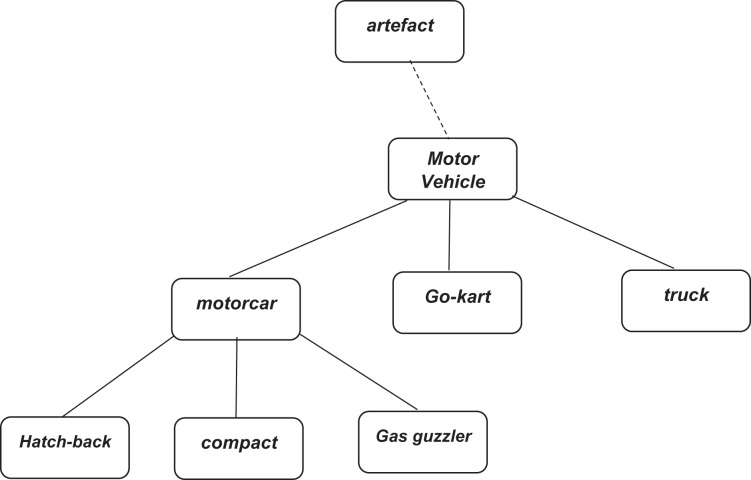
Fragment of WordNet concept hierarchy (https://www.nltk.org/book/ch02.html).

A WordNet is a machine-readable dictionary. It is a lexical database for the English language. In WordNet, nouns, verbs, adjectives, and adverbs are grouped into synonyms set. That set is known as synsets. Each word expresses a distinct concept. These synsets are interlined. There exist semantic relations and linguistic relations between the items of synsets. It works like a thesaurus, but WordNet has an advantage as it groups words with a particular sense. For example, wordNet lexicalized the main concept of “key” by making a synonym set of terms related to the idea. Figure 3 demonstrates an example of a concept hierarchy made by WordNet.

#### Wiktionary

Wiktionary is a web-based project it provides a free dictionary related to the content of terms. It contains data in a semi-structured form. In NLP tasks, Wiktionary offers the opportunity to convert lexicographic data into a machine-readable format.

#### Google search results

Google provides search results related to keywords given by the user in a query. The searched results are usually retrieved based on tokens in the query. The most relevant keyword appears at the top of the page. At the same time, they are arranged in descending order of relevancy. In creating the distractor list, the keywords of Google search results are also used.

### The basic flow of the system

The system works in three significant steps as follows:

- informative sentence extraction
- key identification
- distractor generation

**Figure 4:**
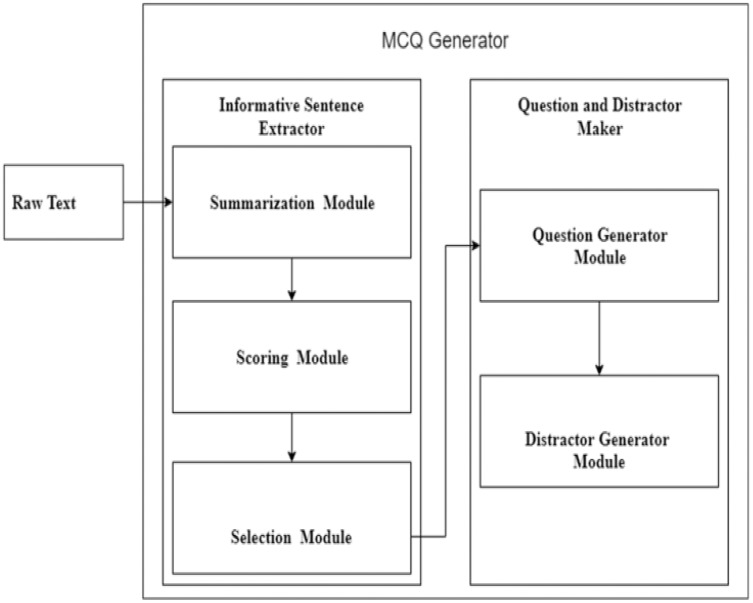
Basic flow diagram of MCQ generation modules.

The steps mentioned above are performed by introducing two modules in the system. The first module is the informative sentence responsible for extracting essential stems from the given unstructured text. It is further divided into three sub-modules summarization, scoring, and selection. In the summarization module, the BERT language understanding model generates a summary. The summary lines are then scored based on features. The lines with a high score are selected as informative. The informative lines are then passed to the stem and distractor generation module that identifies the key from the informative sentences, replaces the answer with blank space, transforms the statement, and generates distractors. The basic flow of the system is shown in Fig. 4.

### Problem description

Currently, most of the assessments are comprised of MCQs. Mostly, it becomes challenging to find the informative sentence, key, and distractor for the generation of MCQs, especially in the computer science domain. Thus, making an intelligent system that could generate MCQs from unstructured text is required.

### Specific objective

The objective of the proposed system are as follows:

- To make a desktop-based application that could generate MCQs from the unstructured text of the computer science domain.

### Scope of the system

The proposed system is able:

- To make the informative sentence-based MCQs of the computer science domain from the given unstructured text.

The rest of the article is organized as follows.

Section 1: A preliminary section discusses the problem, the potential solution, and a concise introduction of techniques and methods used in the proposed system.

Section 2: Presents the literature review for designing an automated system for informative sentence extraction and stem and distractor generation.

Section 3: We discussed the research methodology, including the summarization method, scoring parameters, informative sentence selection method, and stem and distractor generation approach.

Section 4: The discussion and results are provided in this section.

Section 5: The conclusion, restrictions, and future work of the system are presented.

## Background and literature review

For the assessment of students, academic performance assessments are necessary. Assessments provide an idea about the level of subject understanding attained by the student. As assessment could be done at any time, the generation of academic assessment elements should be less time-consuming. MCQs standardize the test while evaluating performance by a different instructor and time taken during the assessment process. MCQ-based tests are conducted on a large scale with less evaluation and assessment time than other evaluations (Correia et al., 2012).

MCQs are commonly the most extensively adopted and constructive type of objective test. MCQ-based assessment is imperative for determining the most significant learning outcomes, understanding, opinion, and analytics. MCQ contains short text as a stem with multiple distractors as options. Distractors include one right or most relevant answer, while other choices refer to wrong or less relevant answers. MCQ consists of three parts. First is the question statement, the second is the key and the third is the distractor’s statement that represents a question. The question may be of two types: fill in the blank based or WH question. ‘WH question’ may start with 5W1H like who, what, where, why, and how, whereas ‘fill in the blank question’ is defined as a statement with a word/term missing from it. MCQ is usually added as a subsection in the objective part of the article. It is a quick way of assessing. Teachers prefer MCQ because it provides ease in marking (Aldabe, Maritxalar & Martinez, 2007). Marks division is also comfortable in the case of MCQ rather than short answer questions. Different students may write the other answers as distractors that are also relevant to the context of the question. Hence, distractors help to know the level of understanding of individual students. However, manual MCQ generation is a time taking task. Reading the whole topic and extracting essential lines or concepts for MCQ generation is challenging. Similarly, making statements short and choosing options are tough. Automatic MCQ generation is getting popular; many tests are being taken online and making the process of MCQ generation fast and efficient (Aldabe & Maritxalar, 2010).

### Purpose of MCQ generation systems

Considering the importance of assessment for quality learning, MCQs based assessment is considered as a quick evaluation technique. Therefore, MCQs are generally adopted for evaluation on a large scale in various domains and applications. In literature, the authors researched on automatic generation of MCQ for different languages and domains like work carried out for the English language, Brown, Frishkoff & Eskenazi (2005), Sumita, Sugaya & Yamamoto (2005) for e-learning, Correia et al. (2010) for the Portuguese language, Aldabe & Maritxalar (2010) for the Basque language, Kurtasov (2013) for the Russian language, Alrehaili et al. (2021) for history, Teo 2020; Afzal & Mitkov (2014) for bio and medical, Fattoh (2014), Effenberger (2015) for a public domain, Goto et al. (2009), Goto et al. (2010), Santhanavijayan et al. (2017) for active learning and e-learning and Majumder & Saha (2014), Majumder & Saha (2015) for sports and entertainment. In literature, an automatic MCQ generation process is carried out in six steps: preprocessing the given text; then, techniques are carried out for sentence selection, key selection, question formation, and distractor generation. In the end, post-processing is carried out to improve the quality of questions.

### Preprocessing

Many researchers performed preprocessing steps to generate MCQs from the text automatically. Preprocessing consists of text normalization, structural analysis, sentence simplification, lexical analysis, statistical analysis, syntactic analysis, coreference resolution, and word sense disambiguation. More precisely, text normalization refers to removing other words from the sentence used by Heilman & Smith (2010). The structural analysis involves adding tags to chapters, and headings are performed in some systems (Chen, Liou & Chang, 2006). Sentence simplification is converting complex and compound sentences to simple sentences (Pino, Heilman & Eskenazi, 2008; Heilman & Eskenazi, 2007). Lexical analysis refers to splitting up the document into words, symbols, and numbers that have been performed in some systems like Heilman (2011), Bednarik & Kovacs (2012). Statistical analysis including counting frequency of words, n-gram frequency, Tf-IDF (Wu et al., 2008) and co-occurrence statistics has been used in Teo (2020). The syntactic analysis involves speech tagging parts, named entity recognition, and generation of parse structure (Aldabe et al., 2006; Karamanis & Mitkov, 2006). Coreference resolution aims mapping to nouns to their concerned nouns is used by Alammari, Sohaib & Younes (2022), Shah (2012). Word sense disambiguation means identifying the word’s exact sense in a given sentence (Liu et al., 2005).

### Sentence selection

The next step is an informative sentence extraction. The stem is a sentence having the questionable fact that may be selected as a candidate for MCQ. Stem selection approaches consist of sentence length, the occurrence of a particular word, parts of speech (POS) information, parse information, semantic information, ML, and summarization. Stem length should not be too lengthy nor too short; this criterion has been used in several works like Aldabe, Maritxalar & Mitkov (2009). The occurrence of a particular word in the sentence is used as a sentence selection technique in Smith, Sommers & Kilgarriff (2008), Smith, Avinesh & Kilgarriff (2010). POS info refers to stem selection based on verb or adjective-noun pair (Lin, Sung & Chen, 2007). Parse information is selection on a parse tree structure, *i.e*., subject-verb-object (Mitkov, Hale & Nikiforos, 2006). Semantic information is noun-pronoun relation based selection including feature extraction and named entity recognition (NER) (Fattoh, 2014), coreference resolution (Lee et al., 2011) and paraphrase detection (Srivastava & Govilkar, 2017). ML is used for sentence selection by using benchmark algorithms. Many authors used ML algorithms, including Naïve Bayes (Hoshino & Nakagawa, 2005), SVM, ranking voted perception, neural networks (Kumar, Banchs & D’Haro, 2015), and counter propagation network-based classification, *etc*.

#### Summarization

For sentence selection, some authors used the summarization technique like (Kurtasov, 2013) and extensive summarizer (Narendra, Agarwal & Shah, 2013), but the methods are outdated now; they did not implement deep learning in summarization. Until recently, recurrent neural networks (RNN) with long short-term memory (LSTM) have been used for many NLP tasks. But these methods do not perform well in the case of lengthy sequences and are prone to overfit even after a lot of tanning, massive computer resources, and many hours of training (Vaswani et al., 2017). Keeping this fact in mind, a better architecture known as Transformer is presented (Vaswani et al., 2017). The Transformer architecture is built using the attention mechanism and feed-forward neural networks (Vaswani et al., 2017). Though, Transformer overcomes many problems that occur while using RNN and LSTM. Later on, at the end of 2018, an unsupervised learning architecture BERT is presented at the top of Transformer architecture (Devlin et al., 2018). BERT is a trained architecture developed by researchers from Google. BERT surpasses several state-of-the-art methods in terms of performance for NLP tasks (Devlin et al., 2018). Since it is pre-trained architecture, it may be used for transfer learning to perform many NLP-related tasks (Barouni-Ebarhimi & Ghorbani, 2007). Prevalent methods lack to provide a dynamically sized summary. But using BERT, a dynamically sized summary may be generated since BERT generates sentence embeddings.

#### BERT for text embedding

As BERT gives outstanding performance than other NLP algorithms, BERT is selected for creating Text Embeddings. BERT is built on Transformer architecture, but its objectives are definite for pre-training. BERT masks out 10% to 15% of random words in the training data; masked words are attempted to be predicted. BERT also takes an input sentence and the candidate sentence to predict whether the candidate sentence follows the input sentence properly (Devlin et al., 2018). This training process is time-taking and requires a lot of computation power. It requires GPUs for training. Keeping this problem for public use, Google released two BERT models. One of these models comprises of 110 million parameters, while the second model includes 340 million parameters (Li et al., 2018; Aljuaid et al., 2021). As the large pre-trained BERT model provides outclass performance, so it is selected for summarization purposes. Using pre-trained BERT, Multiple layers may be chosen for creating embeddings. By using the [cls] layer, the NxM matrix is formed by BERT for clustering purposes. NxM refers to the number of sentences, and M represents embeddings dimensions. It is noticed that embedding representation produced by the [cls] layer is not nearly good. But due to BERT architecture, the tokenized words are equalized by creating NxExM embeddings for the output of other layers in the network, where M in NxExM balanced tokenized words. This issue can be overcome by averaging the embeddings to produce a matrix of order NxE.

#### Clustering embeddings

After completing the embedding from the N-2 layer, the matrix of order NxE is ready for clustering. Sciket learn library is used for the implementation of K-Means clustering. Sentences closest to the centroid are selected as candidate summary sentences.

Considering the background and related work, the gap in existing research and projects was automatic MCQ generation based on the informative sentence. An informative sentence is based on the topic’s core idea or meaningful sentences are extracted by the summarization technique by leveraging the most up-to-date BERT architecture.

#### Keyphrase extraction algorithm

Identifying noun phrases is an essential goal of the keyphrase extraction algorithm (KEA). In-text summarization, text categorization, and information retrieval (IR) systems, KEA plays an important role. KEA identifies phrases based on features like the position of the phrase in the document and the number of occurrences in the document. KEA also classifies the candidate phrase in the document. However, this algorithm sometimes provides incoherent phrases that don’t correspond to the text’s summary (Mishra & Singh, 2011). The KEA is used for both supervised and unsupervised ML tasks (Zhu et al., 2013). To mine high-quality N keyphrase candidates from the given text document, the KeyRank method is presented by Wang, Sheng & Hu (2017). KeyRank algorithm first identifies all phrases from the text. Subsequently, it ranks these candidate phrases, and finally, top-10 N key phrases are extracted from the document. Unlike KEA, KeyRank algorithm performs outclass and provides good results (Wang, Sheng & Hu, 2017). The key phrase extraction algorithm also effectively identifies the number of clusters in a massive number of documents (Han, Kim & Choi, 2007). To mine top-quality keyphrases from the text, Wang, Mu & Fang (2008) presented a technique. The technique was based on the semantic meaning of phrases. The method first extracts the phrases from text and then the semantics of these phrases are checked using the word sense disambiguation technique.

#### TF-IDF

TF-IDF refers to term frequency, inverse document frequency. As the name depicts, this method calculates the relevance of terms in specific documents (Qaiser & Ali, 2018). TF calculates the raw frequency of terms by considering the number of occurrences of that word in the document. In comparison, IDF assigns the weights to the words. To remove stop words, lower weights are assigned to highly frequent terms, and higher weight is given to low frequent words (Sohaib & Olszak, 2021). The attention-based refined TF-IDF method was proposed by Zhu et al. (2019), which identifies hot terms in the document based on time distribution information. TF-IDF is considered as an essential practice for discovering hot terms within the document (Zhu et al., 2019).

### Key selection

The key is the word or phrase to be blanked in MCQ. The literature techniques include frequency count, POS & parse information, semantic information pattern matching, and ML for key selection. Frequency count has been used in Coniam (2013), Shei (2001) instead of the simple term frequency TF-IDF has also been used in Karamanis & Mitkov (2006) and Aldabe, Maritxalar & Mitkov (2009). Semantic information like semantic network structure (Sung, Lin & Chen, 2007), predicate extraction-based approach (Fattoh, 2014), semantic relation among key concepts, word sense disambiguation-based method (Hoshino & Nakagawa, 2007) and property instances in ontology has been used for key selection by researchers. Pattern matching uses similar structural features (Belkin & Croft, 1992; Gates, 2011), and ML for generating verbs, parts of idioms, or adverbs by ML techniques are applied by Curto (2010). While POS and parse information have been used in some works for key selection (Sohaib, Naderpour & Hussain, 2018), some used verbs, adjectives and prepositions (Sohaib, Naderpour & Hussain, 2018) for the key. The proposed system also utilizes POS information for the selection of keys.

### Question formation

Question formation converts a declarative sentence to an interrogative form of questionable form. The researchers used different techniques for question formation. The method for question formation is appropriate Wh-word selection (for example, ‘who’ for people and ‘where’ for location, *etc*. by parse structure) used by Majumder & Saha (2015), by using subject-verb-object (term occurrence, position, and type) operated by Mitkov, Hale & Nikiforos (2006), knowledge-based (by applying knowledge labels to the concept, like ‘what is meant by’ for definition and ‘how do you perform’ for the procedure, *etc*.) used by Pabitha et al. (2014), by syntactic transformation (question based on answers) attempted by Das & Majumder (2017), by discourse connectives (appropriate questions for temporal, casual, result, *etc*.) used by Das, Majumder & Phadikar (2018), semantic information based (semantic role labeling) used by Lindberg et al. (2013), Mazidi & Nielsen (2014), semantic-based transformation (questions based on semantics) proposed by Yao & Zhang (2010). At the same time, some other works made questions by using fill in the blank in it (Bhatia, Kirti & Saha, 2013).

### Distractor generation

Distractors should distract sufficiently. The approaches based on POS information for distractor generation (key and distractor both should be of the same POS) are proposed in Coniam (2013). Frequency (occurrences of both key and distractor should be the same) used in Sohaib, Naderpour & Hussain (2018), domain ontology (by using web ontology language (Antoniou & Van Harmelen, 2004)) proposed by Papasalouros, Kanaris & Kotis (2008), distributional hypothesis (similar words in a similar context) by Celikyilmaz & Hakkani-Tur (2011), pattern matching (by using parse information) used by Hoshino & Nakagawa (2007), by semantic analysis based approach like (Aldabe & Maritxalar, 2010) used latent semantic analysis, (Aldabe, Maritxalar & Mitkov, 2009) used verbs similarity by distributional data, (Belkin & Croft, 1992) used semantic similarity between two words using Patwardhan and Pedersen’s method, Kumar, Banchs & D’Haro (2015) used word2vec tool. Deep semantic analysis and neural embedding-based approaches can be used for sophisticated distractor generation. Using Wikipedia (Krishna et al., 2015), “key” was used as a domain concept for finding the sibling of the key to use them as distractors. At the same time, WordNet (a database for generating synonyms and their relationship, close relation synonyms can be used as distractors) was deployed in Lin, Sung & Chen (2007).

### Post-processing

It is the process of improving the quality of system-generated MCQs. It includes question post-editing, question filtering, and question ranking. Question post-editing includes spelling mistakes, replacement of distractors and rephrasing deployed by Mitkov, Hale & Nikiforos (2006).

### MCQ system evaluation

The automatic MCQ generation system was evaluated based on distracters’ closeness, difficulty, readability, *etc*. As a result of evaluation (Santhanavijayan et al., 2017) scored 72%, and (Majumder & Saha, 2015) scored 93.21% for informative sentences. However, standard evaluation techniques are missing. For computer-generated MCQs, most of the systems adopted manual evaluation of output. There are different metrics for assessing the quality of stem, key, and distractors (Mitkov et al., 2009; Krishna et al., 2015).

#### Evaluation of stem and key

It is observed that the majority of MCQ systems are evaluated by human evaluators as there is no standard dataset publicly available for evaluating automated generated MCQs. For evaluations, evaluators created private data for testing system quality with human evaluators’ help. For stem and key evaluation, sentence length, simplicity of sentence, the difficulty of sentence and key, informativeness of sentence, the sufficiency of context, the difficulty of the key, domain relevancy, grammatical correctness, and correctness of sentence have been used in evaluation metrics. In Table 1, an overview of different systems is presented. The accuracy of the systems is not compared due to different approaches and the unavailability of a benchmark dataset (Shah, Shah & Kurup, 2017; Susanti et al., 2017).

**Table 1:** Evaluation metrics by various researchers.

System	Type of evaluation	Evaluation_metrics	Accuracy
Liu et al. (2005)	Semi-automatic evaluation	Quality of cloze items	Corresponding to input request system generated 66.2%, 69.4%, 60.0% and 61.5% correct sentences.
Aldabe et al. (2006)	Expert language teacher	Quality of questions	More than 80%
Pino, Heilman & Eskenazi (2008)	Five English teachers	Sentence length, simplicity, or difficulty level	66.53%
Teo (2020)	Two biology students	Useful for learning and answerable, or not	Evaluator1: sentence selection 91.66%, key selection 94.16%, distractor selection 60.05% and Evaluator2: sentence selection 79.16, key selection 84.16%, and distractor selection 67.72%.
Bhatia, Kirti & Saha (2013)	Five evaluators having domain knowledge	The difficulty, domain relevance, question information, over-informative or under-informative	Distractors average accuracy 88% and key accuracy 79.4%
Narendra, Agarwal & Shah (2013)	Three evaluators and evaluation guidelines	Informativeness and relevance	The average score of 3.18/4
Kumar, Banchs & D’Haro (2015)	15 human evaluators	Sentence, gap, and distractors are good	Question sentence 94%, gaps 87% and distractors 60%
Majumder & Saha (2015)	Five human evaluators	Quality of questions	Informative sentences 93.21%, key selection 83.03% and distractor quality 91.07%
Shah, Shah & Kurup (2017)	Human tutors	Question acceptance	70.66%
Satria & Tokunaga (2017)	Five English teachers	Quality of questions	65%
Santhanavijayan et al. (2017)	Experimental results and discussions	Efficiency of system	Informative sentences 72%, blank generation 77.6% and distractor generation accuracy 78.8%

#### Evaluation of distractors

The metrics used by various researchers to evaluate distractors are difficulty, readability, closeness to key, and usefulness. Pino, Heilman & Eskenazi (2008) distractors being assessed based on semantic and syntactic points of view. Teo (2020) tested readability and semantic meaning of distractors by substituting the distractors in the gap. Bhatia, Kirti & Saha (2013) defined the scale “good” if at least one of the distractors is close to the key. Table 1 shows the type of evaluation, evaluation metrics adopted by various researchers, and their systems’ accuracy.

## Methodology

### Dataset acquisition

The automatic MCQ generation system is specifically made for helping computer science students at the school or college level in learning until now (the system could further be extended for other domains). The system takes unstructured data from grade 9^th^ and 11^th^ computer science books in chapters. The automatic MCQ generation system learns from the dataset and then provides quality phrases required to extract informative sentences based on its learning. Two types of datasets are being used in this system; one is employed to extract quality phrases, while the other is used to find keys in the question/stem of MCQ. Dataset for quality phrases and to subsequently find the informative sentence is comprised of about 20,583 phrases and is built by using Computer Science books of grade 9^th^ and 11^th^ and technology-based websites. Further, for the key selection module, a dataset of 1,327 keys is built using Computer Science books and technology-based websites (Baig, 2018; Chattha, 2019; https://www.computerhope.com/jargon.htm; https://www.techopedia.com/dictionary).

### Method

**Figure 5:**
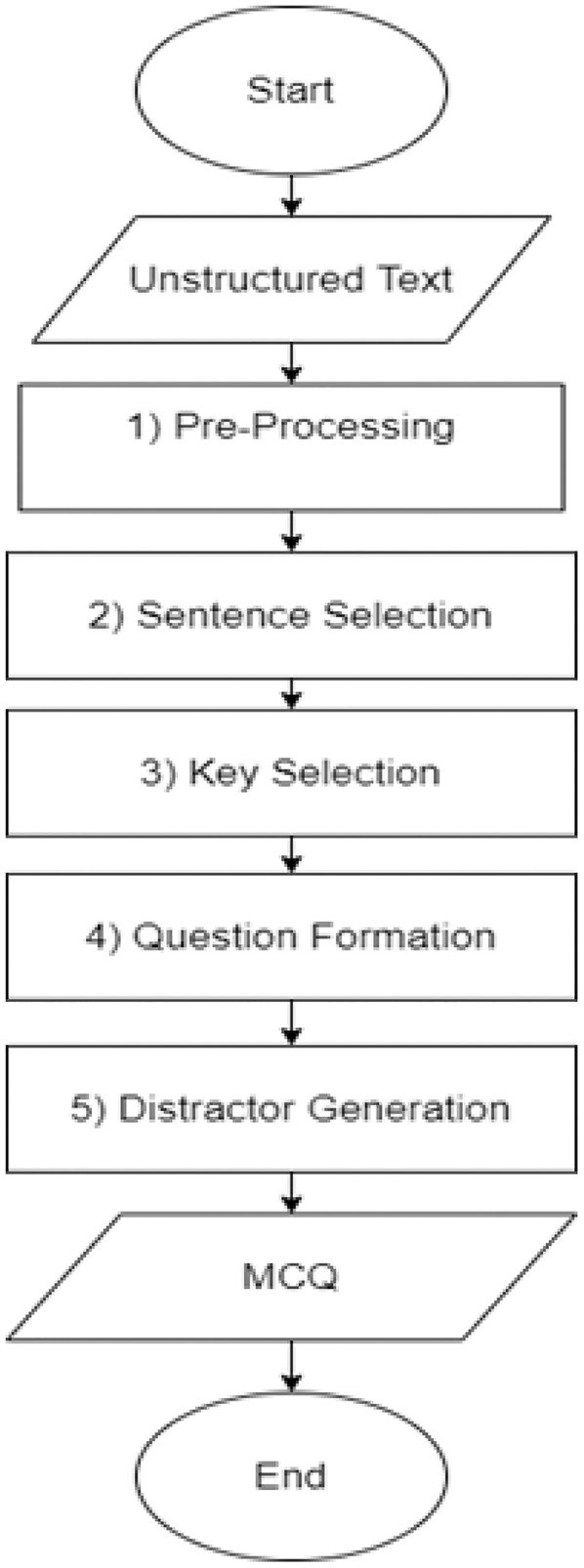
MCQ generation process.

Figure 5 depicts a flow chart of the MCQs generation detailed process. The automatic MCQ generation generic workflow consists of three major steps, informative sentence extraction, key identification, and distractor generation, carried out in the following sequence.

- preprocessing
- sentence selection
- key selection
- question formation
- distractor generation

**Figure 6:**
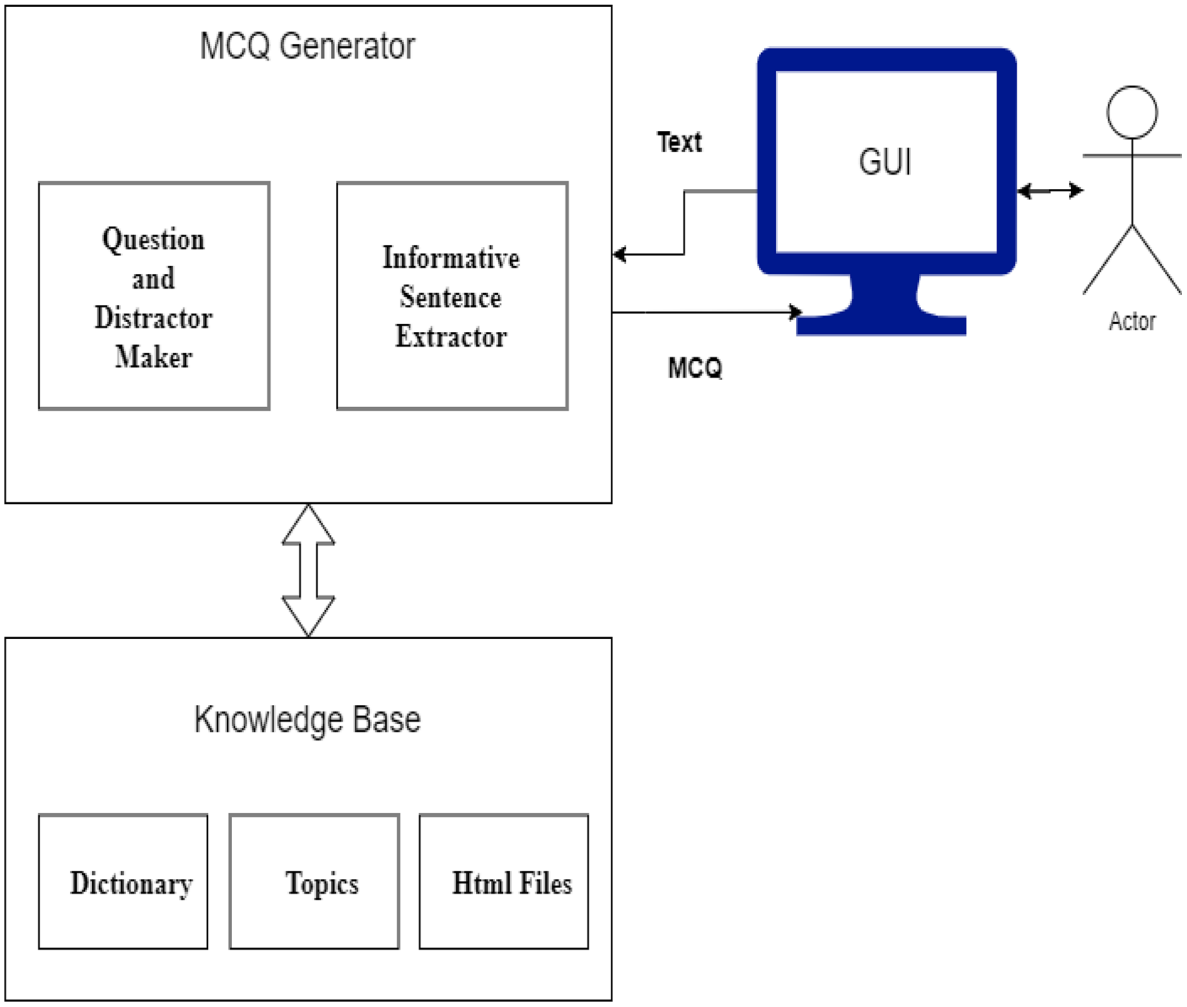
System architecture.

The automatic MCQ generation system provides an interface in which the user provides input in text, and the system provides output in the form of MCQ. The user interacts with the GUI that takes an input, and after preprocessing, informative sentences are revealed using the extractive text summarization technique. The summarized text is scored based on features. The sentences with high scores are then selected for MCQ generation. The candidate sentences then proceed to the stem and distractor generation module. This module contains a knowledge base in the form of dictionaries. Finally, users receive output *via* a GUI-based interface. Figure 6 presents the system architecture diagram; it shows how the user interacts with the system, MCQ generator modules, and modules with the knowledge base.

### Preprocessing

**Figure 7:**
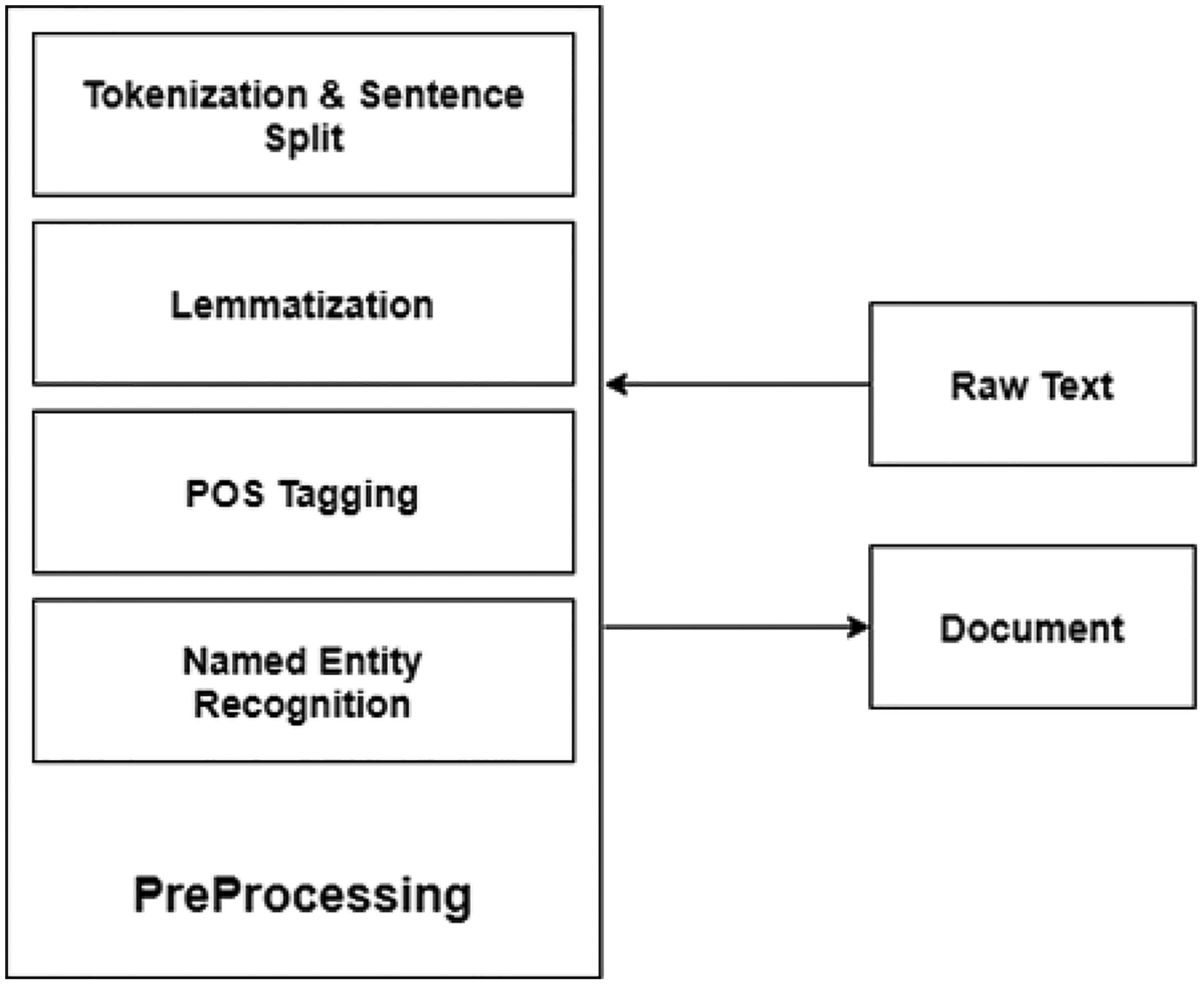
Preprocessing.

At this step, raw text is preprocessed. Preprocessing consists of the following steps using the NLTK toolkit (http://www.tfidf.com/). Figure 7 depicts the preprocessing steps.

- sentence tokenization
- removing special characters
- tokenize words
- remove stop words
- change words in lower case
- lemmatization of words
- repetition of words or frequency of words
- parts of speech tagging
- named entity recognition

### Informative sentence extractor

**Figure 8:**
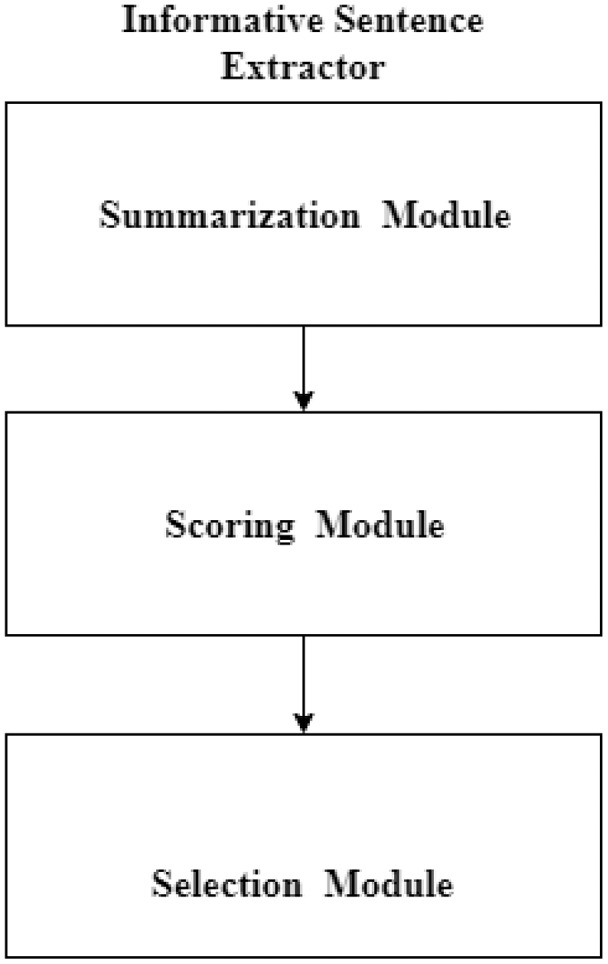
Informative sentence extraction module.

The proposed system aims to select only those sentences for MCQ generation which are informative and most important in the given text. For this purpose, the informative sentence extraction module is further divided into three sub-modules: summarization, scoring, and selection modules. These three modules are responsible for (informative) sentence extraction/selection. Figure 8 shows the sub-modules of the informative sentence extractor module.

#### Summarization module

The summarization module is responsible for creating an extractive summary of input data. In this way, the original text may be reduced up to 50%. In addition, the summary module discards insignificant lines of the input text. We employed a deep learning-based approach, BERT, which creates sentence embeddings for subsequent clustering by employing the K-Means. More precisely, raw text is passed to the BERT model for creating sentence embeddings. The embeddings are then clustered using K-Means clustering, then sentences closest to the centroid are selected as candidate summary sentences. The candidate summary sentences are then scored based on features; the sentences with a high score are chosen for the MCQ generation process.

#### Scoring module

The summarization module creates an extractive summary of input data, thus reducing around 50% of the input text. The output is then given to the scoring module. The scoring module scores all the candidate sentences of summary. The scoring is done based on the features listed in Table 2.

**Table 2:** Scoring features.

Feature	Type	Description
Quality Phrases	Integer	Number of quality phrases in raw text
Average TF	Float	The average frequency of tokens in raw text
Average IDF	Float	Average of the IDF scores of tokens
# of NP	Float	Number of noun phrases in a sentence
# of VP	Float	Number of verb phrases in a sentence
# of Stop Words	Float	Number of stop words in a sentence
# of tokens	Integer	Number of tokens in a sentence
Chapter Title Similarity	Float	Jaccard similarity of a sentence to the title of chapter

#### Quality phrases

The quality phrase mining algorithm is partially automated. It needs to be trained first to generate quality phrases automatically. The computer science domain’s quality phrases are collected from a dataset and verified by domain experts. Figure 8 shows the parameters of quality phrases.

**Figure 9:**
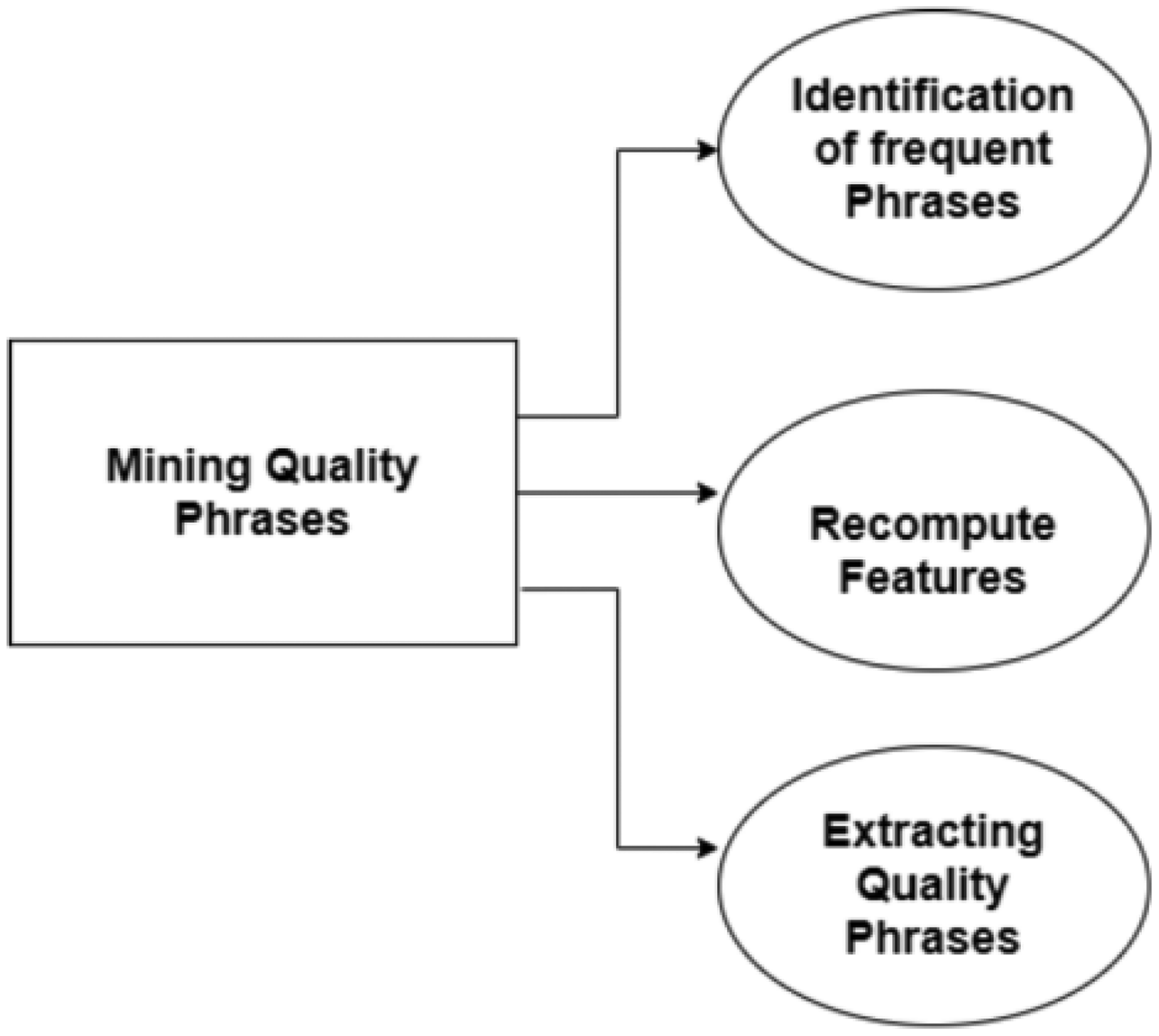
Quality phrase mining.

A phrase is considered as a quality phrase if it possesses the properties of informativeness, completeness, concordance, and popularity (Liu et al., 2015). In addition, a quality phrase must be frequent in a given *corpus*; it cannot be a quality phrase if it is not frequent. A phrase’s quality can be defined as the likelihood of a multi-word series comprising logical and consistent semantic meanings. Suppose if v is a phrase, then Eq. (1) shows the formulation to calculate the quality of phrases.

(1) $${Q(v)}={p(\lceil{v}\rfloor{|}v)}\epsilon\ {[0,1]},$$

where $\lceil{v}\rfloor$ signifies the occurrence of a word in v making up a phrase. If a word is distinct, its quality would be Q(w) = 1. The values between 0 and 1 estimate the phrase or phrase quality (Liu et al., 2015). Figure 9 shows a detailed procedure of quality phrase extraction from the input text.

The details of the features of quality phrases are briefed as follows:

#### Popularity

A quality phrase should frequently appear in the whole given text. A phrase cannot be a quality phrase if it is not occurring with sufficient frequency.

#### Concordance

Identify the sentences with similar meanings. Identification of synonyms is also essential. Equation (2) is used to calculate the concordance feature.

(2) $$p\left( u \right)\; = \displaystyle{{f\left[ u \right]} \over {{\sum _{u^\prime\epsilon u}}f\left[ {u^\prime} \right]}},$$

where u is a word or phrase and u ε U, f[u] is raw frequency, then p(u) shows its probability (Liu et al., 2015).

#### Informativeness

A phrase is informative if it gives information about a specific topic. Equation (3) presents the formula for the calculation of informativeness.

(3) $$IDF\left( w \right)\; = {\rm \; log\; }\displaystyle{{\left| C \right|} \over {\left| {\left\{ {d\epsilon \left[ D \right]\!:\!w\epsilon {C_d}} \right\}} \right|}}$$

Equation (3) calculates the Average inverse frequency of the document. This equation |C| represents *corpus*, d represents a document, and w represents words in a given document (Liu et al., 2015).

#### Completeness

A phrase is said to be complete if it gives full semantic meanings regarding a specific context. Therefore, a phrase should possess completeness to be a quality phrase.

#### TF-IDF

This is another scoring feature used for the proposed system. TF-IDF provides the weight of every word in the given text. This method is numerical statistics (Qaiser & Ali, 2018). TF is the term frequency, which shows how many times a term occurs in a document. IDF gives lesser weight to repeated words and high weight to rare words to remove the stop words from the specified documents (Sohaib & Olszak, 2021). The mathematical Eqs. (4)–(6) are used to compute TF-IDF.

(4) $$t{f_{i,j}}=\displaystyle{{{n_{i,j}}} \over {{\sum _k}{n_{i,j}}}}$$

Equation (4) is used for the calculation of the term frequency of words. In this equation $t{f_{i,j}}$ corresponds to term frequency, of *i* in *j* (http://www.tfidf.com/); where *i* is the word whose frequency is to be computed and *j* is the number of documents having *i*, ${n_{i,j}}$ is the number of instances word *i* emerges in the document and ${\sum _k}{n_{i,j}}$ represents the total number of words in the text.

(5) $$Idf( w )= log\left(\displaystyle{N \over {d{f_t}}}\right)$$

Equation (5) is used to analyze the inverse document frequency of the terms. *N* is the overall number of documents, $d{f_t}$ is the amount of documents containing the term *t*.

(6) $$W_{i,j} = t{f_{i,j}}*{\rm log}\left(\displaystyle{N \over {d{f_t}}}\right)$$

Equation (6) is used to compute the weight of each word in the document. To calculate the weight of words within the text, term frequency multiplies with the inverse document frequency.

#### No. of nouns and verbs

The number of nouns shows the # of nouns and verbs in the sentence.

#### No. of stop words

It shows the number of stop words in the sentence.

#### Jaccard similarity of the title with sentences

The Jaccard similarity of the title of the chapter is compared with each sentence. A sentence having tokens similar to the title gets more scores. Equation (7) depicts the formulation of the Jaccard similarity index.

(7) $$J\left( {A,\; B} \right){\rm \; } = \displaystyle{{\left| {A \cap B} \right|} \over {\left| {A \cup B} \right|}}=\displaystyle{{\left| {A \cap B} \right|} \over {\left| A \right| + \left| B \right| - \left| {A \cap B} \right|}},$$

where A is the set of tokens in the title, while B contains tokens in the sentence.

Each candidate sentence is scored based on scoring features, and a total score (sum of all the scores) is assigned to each sentence as presented in Eq. (8). The output is then sent to the stem selection module for further processing.

(8) $$\rm {Sentence \; Score} = {QPS+TF{-}IDF+CNV+CSW+JS},$$

where, QPS referes to quality phrase score, TF-IDF depicts term frequency-inverse document frequency score, CNV denotes.

#### Sentence selection module

The scoring module assigns a score to each candidate sentence, and the output proceeds to the sentence selection module. Finally, the stem selection module is responsible for selecting top-ranked sentences. This module chooses 20% of top-ranked sentences as candidate sentences for MCQ generation. The selection is made on the aggregated/total score of the sentence. Hence, informative sentences are extracted.

### Stem and distractor generation module

This second module is responsible for the following three tasks: key selection, question formation and distractor generation.

#### Key selection

It is noticed that the selection of keys relevant to the educational context is dependent on human judgment. A dictionary contacting pertinent keys to the computer science domain is built. The key dataset is used for picking the important keyword as a key from a sentence. A dataset of 1,327 keys is made for the key selection module using 9th and 11th-grade books and technology websites (Baig, 2018; Chattha, 2019; https://www.computerhope.com/jargon.htm; https://www.techopedia.com/dictionary). At this step, the system selects a key from the candidate sentence by

- Skimming the sentence
- Finding domain-relevant keys in the sentence with the help of the dataset.

#### Stem formation

Once the key is selected, the stem can be formed by replacing the key with a blank. The underlying module works on the following steps:

1. Scan sentence
2. Select key
3. Replace the key with fill in the blank

#### Distractor generation

The key is given as a sample of input text to the distractor generator for distractor generation. Next, a list of distractors is created using WordNet, Wiktionary, and Google search results. This process includes the following steps for distractor generation.

#### Creating a list of distractors

- By using WordNet, finding synonyms of key
- Finding synonyms on Wiktionary one by one
- Providing derived words of key
- Repeating the process for all synonyms
- Adding all results in a list of dictionary
- Finding list items on Google search
- Picking one item from the list
- Including the “AND” operator as a search query, *i.e*., “Keyboard And.”
- Searching given suggested Google equery one by one
- Scanning results of the searched query for relevant keywords
- Adding discrete effects in a list of dictionary

**Figure 10:**
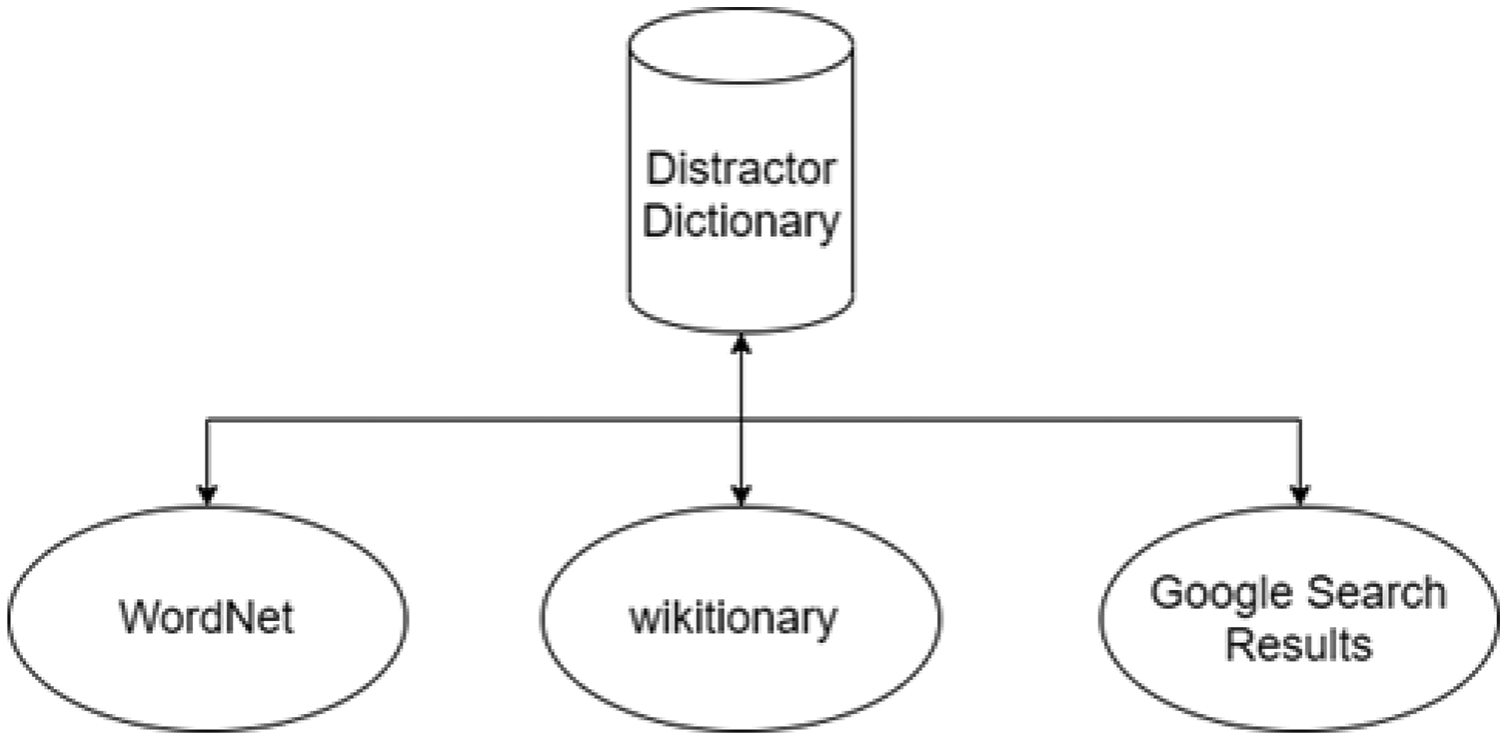
Distractor dictionary.

The system uses online knowledge databases and resources available on the internet for candidate distractor generation. The first step for distractor generation is finding the key synonyms and finding similar or relevant keywords. Three different candidate distractors include synonyms containing a similar or related concept pertinent to the correct answer. Next, network-accessible encyclopedias like Wiktionary and Google search results are used for selecting distractors. After generating the list of distractors, the next step is the selection of distractors. Then, randomly three distractors are chosen from the list. The distractor dictionary diagram is shown in Fig. 10.

### System design and implementation

The automatic MCQ generation system comprises of two components:

#### Front end

A desktop application is designed that provides an integrated development environment to the user. The user inputs a raw text for the interface, and as a result, the output is shown on the desktop app, and a “.txt” file is also created containing all the output of the given input, *i.e*., MCQs.

#### Back end

**Figure 11:**
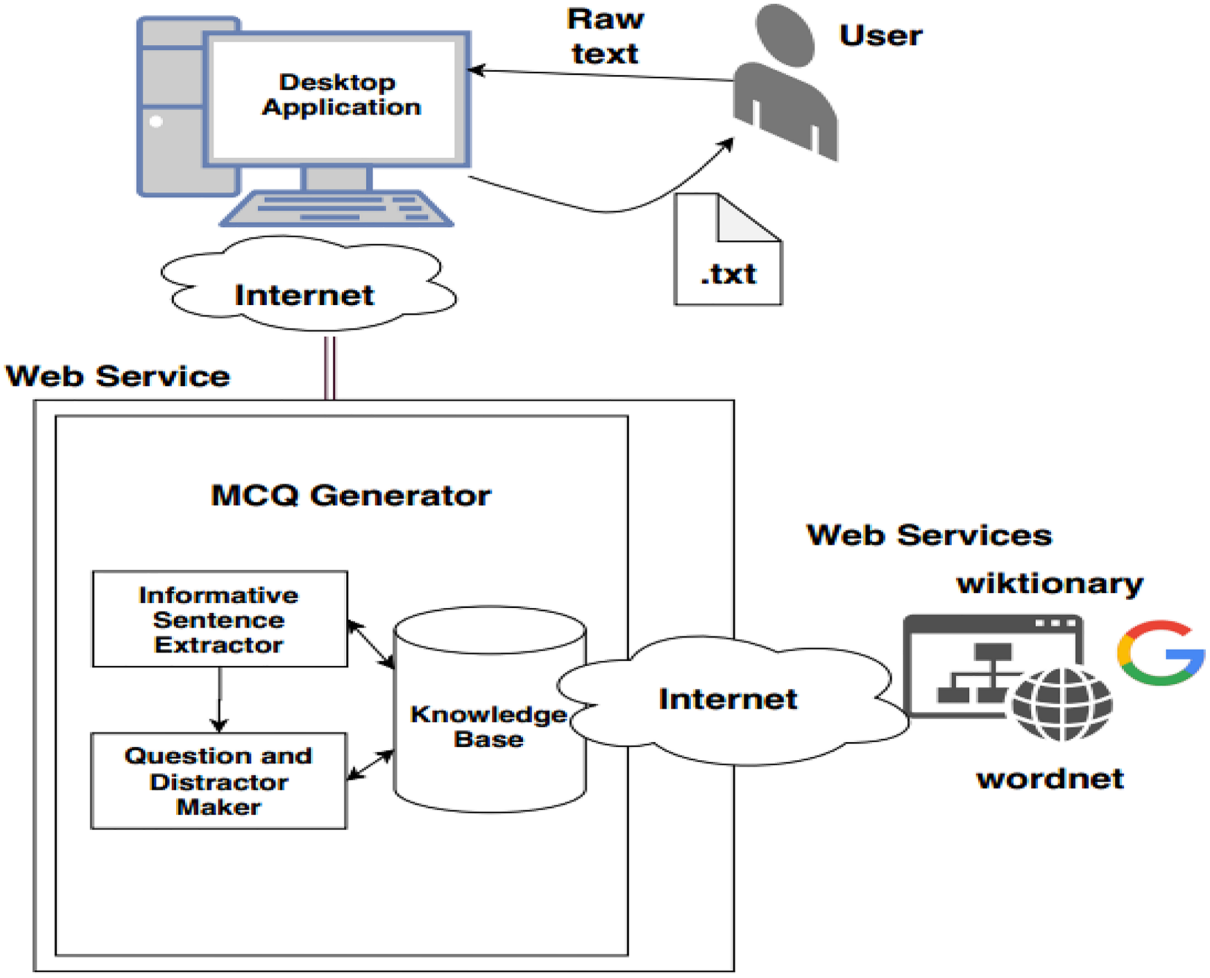
System architecture.

All the backend files of the system reside on cloud service. The desktop application retrieves the functionality of the backend system and provides the results to the user. System architecture components are presented in Fig. 11.

#### System requirements

Automatic MCQ generation system based on two components front end and back end. The system’s back end resides on the AWS cloud, while the front end comprises of a desktop application that runs on the user terminal. The specifications of the server and terminal are given in Table 3.

**Table 3:** System requirements.

Sr#	Description	Detail
1	Server Platform	Ubuntu
2	Server RAM	8 GB
3	Server Storage	10 GB
4	Server CPUs	2 vCPU
5	Terminal Platform	Ubuntu/Windows
6	Terminal RAM	4 GB
7	Terminal Storage	2 GB

## Results and discussion

This section includes the results of all the procedures through which raw text passed. All the steps are discussed in detail in Section III. Here the results of the system are discussed. For this purpose, the data set used for testing is taken from a 9th and 11th grade computer science book consisting of five and 10 chapters, respectively. Each chapter comprises of several subtopics in it. For testing purposes, the full one chapter’s unstructured text is given as input at a time.

### Providing unstructured text

**Figure 12:**
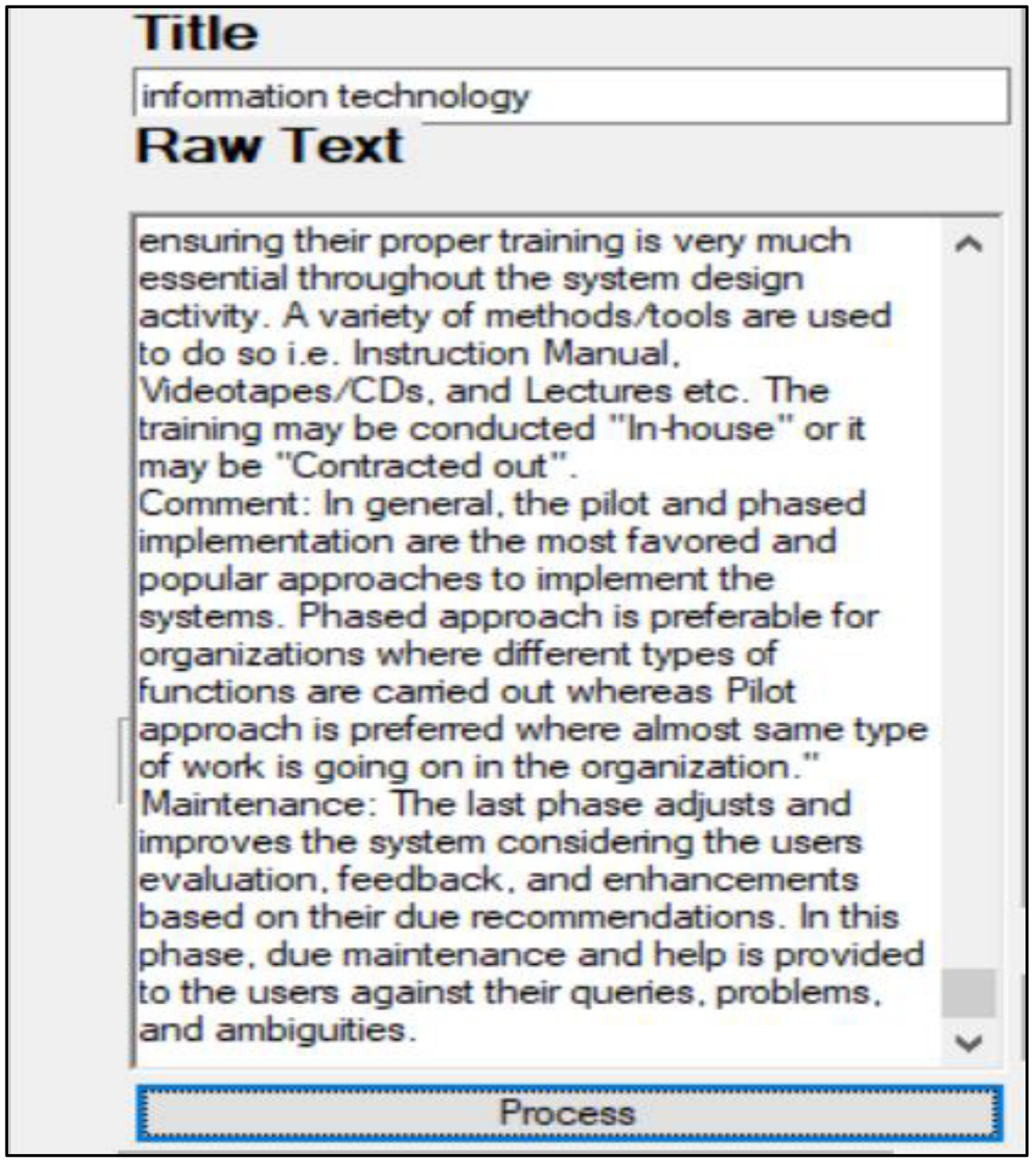
Desktop app input fields.

The user first writes the title of a chapter in the “Title” field of the interface and unstructured text in the “Raw text” field. Then, after pressing the “Process” button, the processing is started. Figure 12 shows the input fields of the desktop interface, taking the raw text as input.

### Processing of text

At this step first BERT model creates embeddings of all the sentences. Then, the k-Means clustering algorithm makes clusters, and in this way, sentences close to the centroid are selected for further processing. The next step is the scoring of candidate sentences. Finally, each sentence is scored based on scoring features.

### MCQs generated by the system

**Figure 13:**
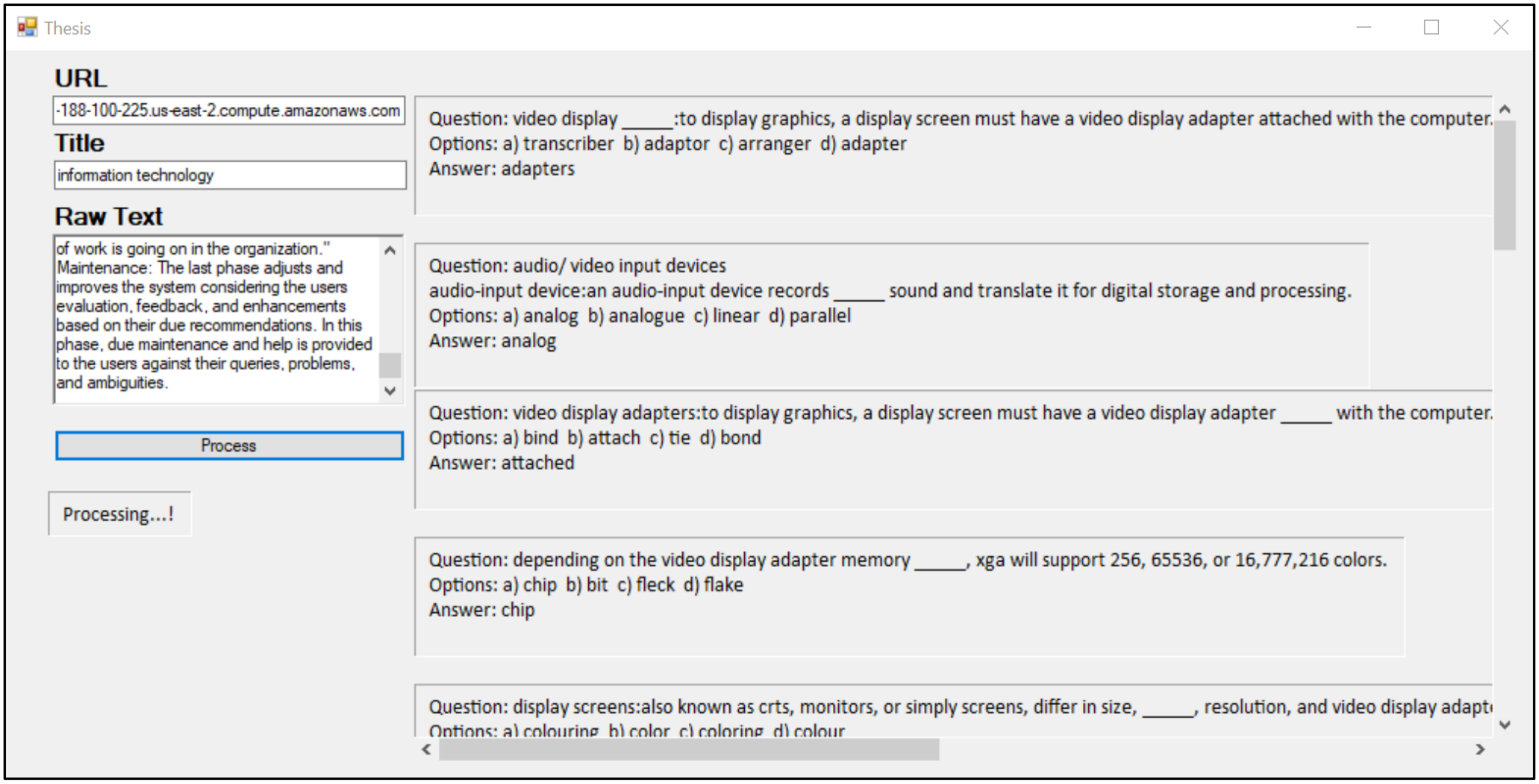
Desktop app full view.

After scoring, 20% of the sentences with a high sum score are selected as MCQ candidates. A full view of the desktop app can be seen in Fig. 13.

### Evaluation of the system

Ten domain experts evaluated the system. In addition, domain experts assessed the system’s efficiency on informative sentences, key generation, and distractor generation parameters. Therefore, the results of the system are presented in Table 4.

**Table 4:** System evaluation results by domain experts.

	Informativeness	Key generation	Distractor generation
Evaluator 1	8.5	7.5	6.5
Evaluator 2	7.5	4	9.5
Evaluator 3	8.5	9.5	9
Evaluator 4	9.5	8	7.5
Evaluator 5	8.5	7.5	8.5
Evaluator 6	9	9.5	9
Evaluator 7	8.5	6	7.5
Evaluator 8	8.5	9	9.5
Evaluator 9	7.5	6.5	5.5
Evaluator 10	7	9.5	7.5
Percentage	83	77	80

The proposed system scored 83% for informativeness, 77% for blank generation, and 80% for distractor generation. The overall accuracy of the system is 80%.

## Conclusion and future work

Continued feedback is required for student’s practical learning. MCQs play an essential role in students’ constructive education. Manual MCQ generation involves a lot of effort, time, and domain knowledge. We have presented a system that generates MCQs automatically using computer science domain text as input. As all the sentences are not capable of the generation of MCQs, the automatic MCQ generation is carried out by following three steps; the first step is the extraction of informative sentences, the second step is the identification of the key, and the third step is determining the distractors relevant to the key. We propose a novel method involving NLP and ML techniques for the generation of MCQs. The preprocessing of input text *corpus* is performed by NLP techniques like tokenization, lemmatization, POS, *etc*. Subsequently, the proposed method extracts informative sentences by extractive text summarization using the BERT model for creating text embeddings and K-means clustering for getting sentences closest to the centroid for generating a summary. The unsupervised machine learning approach has been used in summarization due to the absence of a human-generated computer science summary labeled data set. Scoring of sentences is done on parameters like quality phrases, TF-IDF, the number of nouns/verbs, stop words, the number of tokens, Jaccard similarity of title, and then sentences with high scores are selected for MCQ generation. For key identification, the knowledge-base is used. The knowledge base contains essential and domain-relevant keywords. It is due to the lack of dataset for key identification, the knowledge-base is made by using computer science books and the web.

Moreover, WordNet, Wiktionary, and Google search results are incorporated for the distractor generation process. Domain experts validated the accuracy of automatically generated MCQs as 80%. Experimental results demonstrated that the proposed method is quite accurate. Finally, the system provides a user-friendly interface that inputs raw text, processes it, and gives MCQs as output. Students, as well as teachers, may easily use this Desktop app to generate MCQs automatically.

### Advantages and assumptions

Some advantages and disadvantages of the research work should be considered to improve future work.

#### Advantages

Machine deep learning techniques used in the system help to achieve the followings:

- Fast and efficient results
- Free of bulky computation devices
- Bettering learning process
- Easily accessible

#### Achieving research objectives

- We can reduce the research gap
- MCQs are based on informative sentences
- These reduce the cost and time of finding informative sentences, keys, and appropriate distractors.

#### Assumption

An assumption must be considered along with the improvement of the proposed system. First, a high-speed internet connection is required for using the system smoothly without any disturbance.

#### Future work

This system may further be improved by introducing abstractive summarization techniques. This system can also make MCQs of other domains by enhancing the dataset of keys and quality phrases. The method for distractor selection may be improved to make more confusing or difficult distractors. Further work can be done on the front end of this system by providing options for the number of required MCQs.

## Supplemental Information

10.7717/peerj-cs.1010/supp-1 Supplemental Information 1 Raw data.

10.7717/peerj-cs.1010/supp-2 Supplemental Information 2 Code.

## Additional Information and Declarations

### Data Availability

The following information was supplied regarding data availability: The code and data are available in the Supplemental Files.
